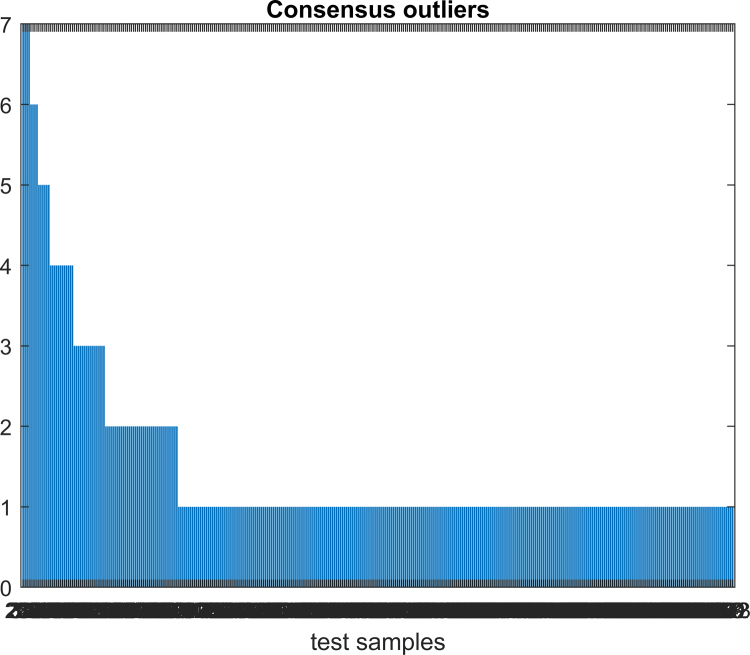# QSAR ligand dataset for modelling mutagenicity, genotoxicity, and rodent carcinogenicity

**DOI:** 10.1016/j.dib.2018.01.077

**Published:** 2018-02-02

**Authors:** Davy Guan, Kevin Fan, Ian Spence, Slade Matthews

**Affiliations:** Pharmacoinformatics Laboratory, Sydney Medical School, The University of Sydney, Australia

## Abstract

Five datasets were constructed from ligand and bioassay result data from the literature. These datasets include bioassay results from the Ames mutagenicity assay, Greenscreen GADD-45a-GFP assay, Syrian Hamster Embryo (SHE) assay, and 2 year rat carcinogenicity assay results. These datasets provide information about chemical mutagenicity, genotoxicity and carcinogenicity.

**Specifications Table**

**Table:** 

Subject area	*Computational Chemistry*
More specific subject area	*Quantitative Structure-Activity Relationship (QSAR) modelling*
Type of data	*Raw data (CSV files), processed data (ARFF files) with analysis*
Data format	*SMILES structures and bioassay results, selected descriptors*
Experimental factors	*Data was gleaned for QSAR model development*
Experimental features	*QSAR models were developed for each dataset using machine learning algorithms using calculated structural descriptors*
Data source location	*Discipline of Pharmacology, Blackburn Building, University of Sydney, Australia*
Data accessibility	*Raw and processed data are presented as CSV and ARFF files, respectively, as supplementary data for this article*

**Value of the data**

• This article contains the largest public collection of ligands and results for the GreenScreen GADDα-45 and Syrian Hamster Embryonic Cell Transformation assays collated from previous literature to date.
• A benchmark dataset of pharmaceutically relevant ligands for use in rat carcinogenicity QSAR models is presented and compared with ligands from regulatory domains.
• Physiochemical descriptors were calculated from the SMILES structures and selected for QSAR model performance.

## 1 Data

The creation of a QSAR model for the 2-year rodent carcinogenicity bioassay is highly desirable since it is the gold standard for assessing potential chemical carcinogenicity. However, previous modelling efforts have been hampered due to data availability and reliability issues stemming from bioassay limitations such as low throughput, high cost, and modest reproducibility between laboratories and rodent species. The *in vivo* carcinogenicity datasets in this article are solely rat carcinogenicity outcomes due to previous literature finding the rat carcinogenicity bioassay produces better endpoint reliability in comparison to the mouse carcinogenicity bioassay. This article presents two rat carcinogenicity datasets from the regulatory toxicology and pharmaceutical safety chemical domains.

Genotoxicity occurs from chemicals acting with genomic mechanisms of toxicity and this has been associated with potential carcinogenicity. This endpoint type features many *in vitro* bioassays with larger libraries of screened molecules in comparison to *in vivo* rodent carcinogenicity bioassay data. QSAR models capable of utilizing this data in combination with rodent carcinogenicity data may address the limited applicability domain of the *in vivo* data. The data was exhaustively collated from the *in vitro* GreenScreen GADDα-45 and Syrian Hamster Embryonic bioassays from the literature. Previous literature found concordance between these bioassays and *in vivo* rodent carcinogenicity outcomes. The Ames Bacterial Mutagenicity Benchmark Dataset has also been included for comparison.

## 2 Experimental design, materials and methods

### 2.1 Dataset preparation

ISSCAN: 854 chemical database of *in vivo* rat carcinogenicity from [1].

PHARM: *in vivo* rodent carcinogenicity results on pharmaceutical chemicals from [2].

GreenScreen: 1415 GADD-45a-GFP assay results from [3], [4], [5], [6], [7], [8], [9], [10].

Syrian Hamster Embryonic: Data on 1415 chemicals extracted from [11], [12].

Ames: 6512 Ames results from [13].

### 2.2 Dataset curation

SMILES structures were generated using ChemAxon JChem for Office from CAS Numbers or chemical names.

These structures were curated using ChemAxon Standardizer to remove salts and solvents and aromatized.

### 2.3 Descriptor selection

The CfsSubsetEval algorithm [14] selected subsets of structural descriptors (generated by PaDEL Descriptor [15]) for each dataset.

### 2.4 Applicability domain quantification

The applicability domain of each dataset compared to the PHARM dataset was quantified using leverage, Euclidean distance from centroid, and a variable *k*nn-based distance [16] measures.

### 2.5 QSAR model Attribute Importance Scores

**Fig. 1:**
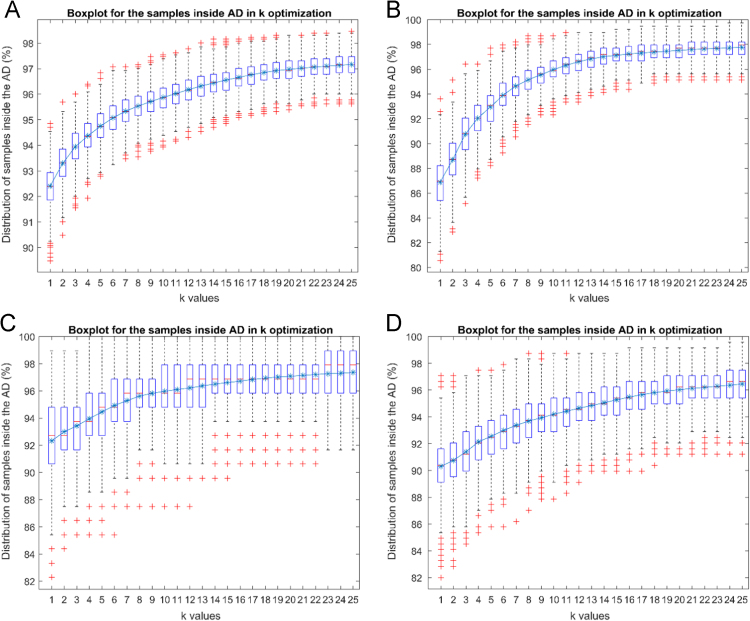
Box and whisker plots depicting the percentage of PHARM validation dataset samples retained within the applicability domain of the processed Ames (A), GSX (B), SHE (C), ISC (D) modelling datasets over different *k* values. *k* values in the variable *k*nn-based distance measure were optimized using 20% of samples in the PHARM validation dataset, 1000 iterations, and a maximum *k* value of 25.

Attribute Importance Scores were calculated from each RandomForest QSAR model (Fig. 1, Table 1, Table 2, Table 3, Table 4, Table 5, Table 6, Table 7).

**Table 1:** Summary of initial datasets after curation.

Name/source	Bioassay/data source	*n*	+/− Results	% Balance (+/−)
Ames Mutagenicity	Ames Bacterial Mutagenicity Benchmark Dataset	6512	3503/3009	54:46
Syrian Hamster Embryonic	Syrian Hamster Embryonic Cell Transformation Assay (pH 7)	356	232/124	65:35
GreenScreen	GADDα-45 GFP Literature Results	1415	163/1252	12:88
ISSCAN	ISSCAN expert rat carcinogenicity calls	854	510/344	60:40
PHARM	Rat carcinogenicity literature results	374	134/240	36:64

**Table 2:** Summary of modelling datasets after structural descriptor calculation and selection.

Name	# Desc.	*n*/Desc
Ames	90	72.4
GSX	21	93.1
SHE	58	8.28
ISC	51	23.5
PHARM	1875	–

**Table 3:** Applicability Domain (AD) quantification comparing each processed QSAR modelling dataset to the PHARM dataset.

Name	AD measure	Inside AD	Outside AD
Ames	*k*nn distance	350	24
	Distance from centroid	356	18
	Leverage	351	23
GSX	*k*nn distance	355	19
	Distance from centroid	353	21
	Leverage	352	22
SHE	*k*nn distance	357	17
	Distance from centroid	356	18
	Leverage	331	43
ISC	*k*nn distance	325	49
	Distance from centroid	330	44
	Leverage	309	65

**Table 4:** Attribute Importance Scores (AIS) and node membership for structural descriptors in the GreenScreen (GSX) QSAR model.

Structural descriptor	AIS	Node membership
AATS2m	0.37	20,529
naAromAtom	0.35	15,136
GATS2m	0.35	17,920
GATS2i	0.35	16,693
SpMin1_Bhp	0.34	15,952
ETA_BetaP	0.32	14,229
IC3	0.3	14,615
nAcid	0.3	4138
nAtomLC	0.29	11,238
nssO	0.29	9829
MDEC-33	0.29	12,339
SHBint6	0.29	7607
R_TpiPCTPC	0.28	12,038
RDF50m	0.28	7741
MPC4	0.28	13,116
L3m	0.27	7252
De	0.26	7117
nHAvin	0.23	3773
MDEN-13	0.21	4086
n3HeteroRing	0.18	1316

**Table 5:** Attribute Importance Scores (AIS) and node membership for structural descriptors in the ISSCAN (ISC) QSAR model.

Structural descriptor	AIS	Node membership
ALogp2	0.4	13,058
AATSC0p	0.38	11,714
ATSC7e	0.38	9518
MATS3s	0.37	11,996
BCUTp-1l	0.37	10,779
MATS1e	0.37	11,671
BCUTw-1h	0.35	9596
nN	0.35	6494
nAcid	0.34	1640
nCl	0.34	3703
P1s	0.33	7280
C3SP2	0.3	4817
nHBint4	0.3	2765
nHBint2	0.29	3028
nssCH2	0.29	5294
topoShape	0.29	5618
nHBint3	0.29	3033
nHBint5	0.28	2579
nHBint6	0.28	1998
C1SP2	0.28	4941
nHCsatu	0.27	3117
nAtomLAC	0.26	3733
ndO	0.26	4003
minsOH	0.26	3633
maxsOH	0.26	3248
nssO	0.26	2945
nsssCH	0.25	3217
maxsNH2	0.25	3572
maxssO	0.25	3885
nssssC	0.25	1917
ndssC	0.25	3828
nBase	0.25	1811
ETA_Beta_ns_d	0.25	3798
SaaS	0.24	612
nHssNH	0.24	1971
n6Ring	0.24	4471
n6HeteroRing	0.23	1761
mindsN	0.23	1969
nHBDon	0.23	3556
nsssN	0.23	2034
LipinskiFailures	0.23	1479
nsOm	0.23	1527
maxdsN	0.22	1748
n5HeteroRing	0.21	1658
MDEN-23	0.2	2373
SRW5	0.19	2445
mintsC	0.19	641
SaaO	0.18	789
MDEN-13	0.18	706
nT7Ring	0.15	574
nF11HeteroRing	0.13	393

**Table 6:** Attribute Importance Scores (AIS) and node membership for structural descriptors in the Syrian Hamster Embryonic (SHE) cell transformation assay QSAR model.

Structural descriptor	AIS	Node membership
ATSC0e	0.39	2283
AATSC4m	0.39	2176
AATS0p	0.38	2522
AATS7i	0.38	1848
MATS2c	0.37	2105
AATS1m	0.36	2582
ATSC6s	0.36	2284
ATSC8s	0.36	1687
GATS2c	0.35	1825
AATSC7m	0.34	1812
MATS7m	0.34	1589
MATS8c	0.33	1571
MATS7p	0.33	1573
GATS1c	0.33	2219
GATS4m	0.33	1943
VE3_Dzp	0.32	2041
SpMin8_Bhp	0.32	1655
nAcid	0.31	448
GATS8v	0.31	1067
GATS8c	0.31	1464
SpMin7_Bhs	0.3	1857
VR2_Dt	0.3	1707
GATS8i	0.29	1172
naasC	0.29	808
TDB2i	0.29	1128
C1SP2	0.29	760
nsCH3	0.29	974
TDB8u	0.28	672
minHBint3	0.28	803
C1SP3	0.28	992
TDB4e	0.27	1057
ASP-6	0.27	1806
nRotBt	0.27	1127
SCH-5	0.27	594
Ds	0.27	972
SHsOH	0.27	897
RNCG	0.27	1039
E3p	0.27	915
RDF10m	0.27	961
TDB1m	0.27	1203
piPC9	0.27	1033
TDB3i	0.26	1314
nsNH2	0.26	400
nHsNH2	0.26	496
RotBFrac	0.26	1203
nssO	0.25	565
minsssN	0.25	376
nHeteroRing	0.25	490
TDB7r	0.24	835
maxssO	0.23	743
SRW5	0.23	442
minHBint7	0.23	424
nssssNp	0.23	25
nAtomLAC	0.22	871
MDEN-12	0.21	208
nT6HeteroRing	0.21	384
nHCHnX	0.2	172
nFG12HeteroRing	0.17	258

**Table 7:** Attribute Importance Scores (AIS) and node membership for structural descriptors in the Ames QSAR model.

Structural descriptor	AIS	Node membership
AATS4e	0.42	17,373
ATSC7c	0.42	15,115
ATSC2c	0.41	18,428
AATS2e	0.41	16,488
AATS1m	0.41	16,634
ATSC4m	0.4	16,224
ATSC3m	0.4	16,652
ATSC2v	0.38	15,374
ATSC3e	0.38	15,359
ATSC4i	0.37	14,988
ATSC2e	0.37	15,187
AATSC5c	0.37	14,636
ATSC2i	0.36	14,319
MATS6c	0.36	13,289
nAcid	0.36	2038
MATS4c	0.36	15,264
ATSC1e	0.35	15,795
MATS4m	0.35	13,467
ATSC1i	0.35	14,449
MATS6i	0.34	12,963
GATS3c	0.34	13,301
GATS1c	0.33	14,472
GATS8m	0.33	9386
AATSC1i	0.32	13,724
AATSC1e	0.32	13,859
GATS5v	0.32	12,664
VE1_Dzp	0.32	11,795
GATS3m	0.32	12,529
MATS1e	0.31	12,967
MATS1i	0.3	12,082
BCUTc-1l	0.3	9751
GATS1m	0.3	12,484
SpMax1_Bhv	0.29	13,212
GATS2e	0.29	12,634
SpMin1_Bhp	0.29	13,295
GATS1p	0.29	11,559
GATS1i	0.29	11,251
SpMax1_Bhi	0.28	12,842
ASP-2	0.28	8374
ETA_EtaP	0.28	11,891
mindsssP	0.27	299
BCUTw-1h	0.27	8210
BCUTw-1l	0.27	5500
hmax	0.27	12,581
BIC2	0.27	11,166
TDB9u	0.27	4470
Mpe	0.27	9879
Du	0.27	6788
ETA_Epsilon_1	0.27	9185
MIC2	0.26	10,461
nHCsats	0.26	4146
MDEC-12	0.26	5914
ETA_Eta_L	0.26	11,100
E3i	0.26	6433
JGT	0.26	10,771
TDB8i	0.26	5577
RDF40m	0.26	7058
ETA_Epsilon_4	0.25	9370
SHCsatu	0.25	4686
BIC1	0.25	11,006
MLFER_S	0.25	10,748
R_TpiPCTPC	0.25	11,100
ETA_dEpsilon_A	0.25	8641
RDF20m	0.25	7793
WTPT-5	0.25	9438
C3SP3	0.25	1330
piPC10	0.25	7381
SaaaC	0.25	4592
RDF20s	0.25	7448
L3u	0.24	7228
SdCH2	0.24	705
ETA_BetaP_ns_d	0.24	5993
AMW	0.24	8526
MLFER_A	0.23	7826
nAtomP	0.23	7347
minHssNH	0.22	3200
ETA_Shape_X	0.22	2980
MDEN-33	0.21	990
mindsN	0.21	2570
minsNH2	0.21	3578
SRW9	0.2	4461
MDEN-23	0.2	2559
maxHCHnX	0.2	2082
minwHBd	0.19	1599
nTG12Ring	0.19	1527
nFG12Ring	0.19	1633
SaaS	0.18	760
MDEN-13	0.17	979
SCH-3	0.15	1699
VCH-3	0.14	1762

## Figures and Tables

**Fig. S1:**
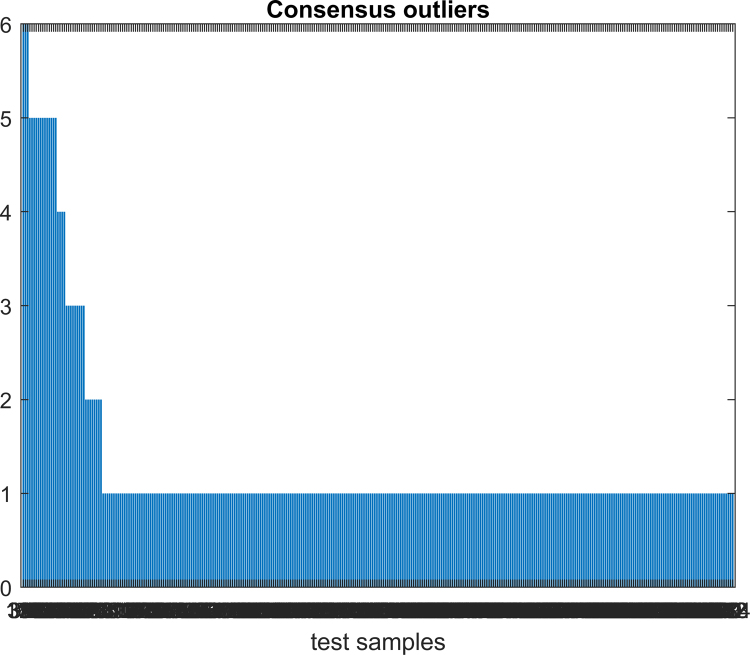


**Fig. S2:**
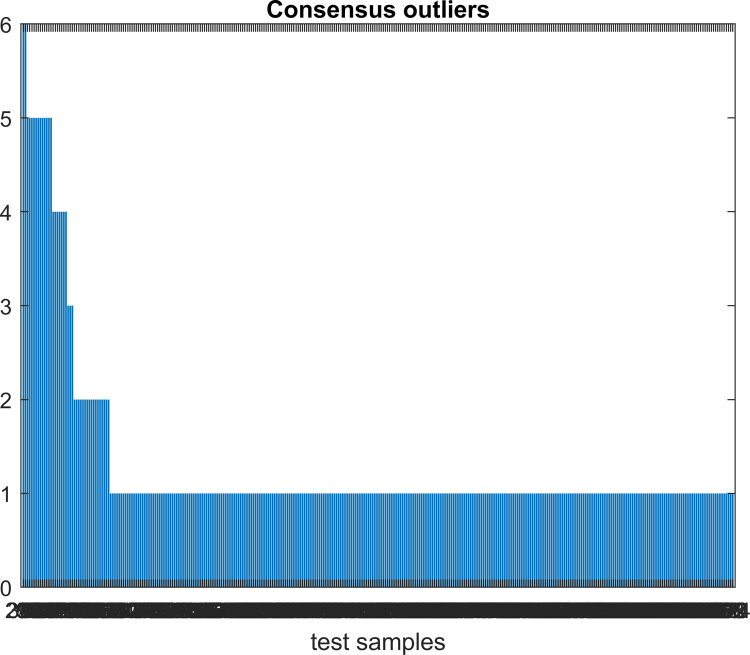


**Fig. S3:**
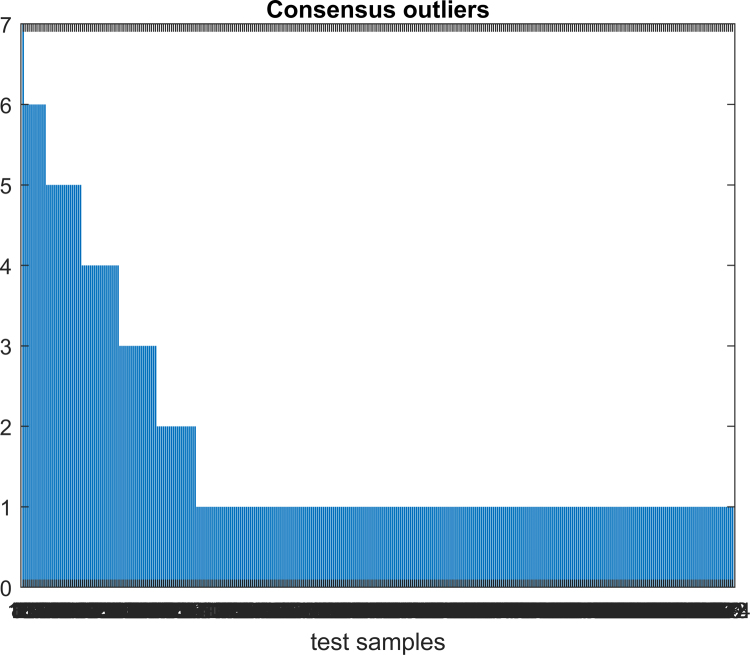


**Fig. S4:**